# Phases and rates of iron and magnetism changes during paddy soil development on calcareous marine sediment and acid Quaternary red-clay

**DOI:** 10.1038/s41598-017-18963-x

**Published:** 2018-01-11

**Authors:** Laiming Huang, Xiaoxu Jia, Ming’an Shao, Liumei Chen, Guangzhong Han, Ganlin Zhang

**Affiliations:** 1 Key Laboratory of Ecosystem Network Observation and Modeling, Institute of Geographic Sciences and Natural Resources Research, Chinese Academy of Sciences, Beijing, 100101 China; 2 State Key Laboratory of Soil and Sustainable Agriculture, Institute of Soil Science, Chinese Academy of Sciences, Nanjing, 210008 China; 3 College of Resources and Environment, University of Chinese Academy of Sciences, Beijing, 100049 China; 4 State Key Laboratory of Soil Erosion and Dryland Farming on the Loess Plateau, Institute of Soil and Water Conservation, CAS & MWR, Northwest A & F University, Xianyang, China; 5 College of Resources and Environment, Zunyi Normal College, Zunyi, 563002 China; 6 College of Resources and Environmental Sciences, Neijiang Normal College, Neijiang, 641112 China

## Abstract

Dynamic changes in Fe oxides and magnetic properties during natural pedogenesis are well documented, but variations and controls of Fe and magnetism changes during anthropedogenesis of paddy soils strongly affected by human activities remain poorly understood. We investigated temporal changes in different Fe pools and magnetic parameters in soil profiles from two contrasting paddy soil chronosequences developed on calcareous marine sediment and acid Quaternary red clay in Southern China to understand the directions, phases and rates of Fe and magnetism evolution in Anthrosols. Results showed that paddy soil evolution under the influence of artificial submergence and drainage caused changes in soil moisture regimes and redox conditions with both time and depth that controlled Fe transport and redistribution, leading to increasing profile differentiation of Fe oxides, rapid decrease of magnetic parameters, and formation of diagnostic horizons and features, irrespective of the different parent materials. However, the initial parent material characteristics (pH, Fe content and composition, weathering degree and landscape positions) exerted a strong influence on the rates and trajectories of Fe oxides evolution as well as the phases and rates of magnetism changes. This influence diminished with time as prolonged rice cultivation drove paddy soil evolving to common pedogenic features.

## Introduction

Iron (Fe) is the fourth most abundant element in Earth’s crust (6.7 wt %)^1^ and serves as an essential micro-nutrient for almost all living organisms^2^. It has a variety of different forms in soil systems, such as primary silicate minerals, pedogenic clay minerals, Fe (oxyhydr)oxides with different degrees of crystallinity, as well as in organic complexes^3,4^. These different forms of Fe are present at variable levels of concentration within soil profiles, during different stages of soil formation, and in different bioclimatic regions due to variations in weathering intensity and soil forming processes^4,5^. The dynamic changes in Fe species and bioavailability in response to shifting redox conditions trigger many processes in terrestrial ecosystems, such as mineral weathering, nutrient cycling, and contaminant mobility^4^. In addition, Fe cycling and partitioning between different mineral and organic forms have been confirmed to play a critical role in the preservation of organic carbon in soils and sediments^6,7^. Thus, a better understanding of the mechanisms and processes that control the temporal and spatial changes of Fe species, amounts and bioavailability in soils is not only important for uncovering element biogeochemistry, but also crucial in fully assessing ecosystem function and service.

Previous studies have shown that Fe (oxyhydr)oxides undergo significant changes in species, amounts, and stability during the course of soil development^4,8–15^. As a result, soil magnetic properties vary markedly with pedogenic time. Magnetic enhancement during natural pedogenesis under aerobic conditions has been widely recognized^16–26^, which was attributed to the formation of ferrimagnetic minerals by different processes, including burning of vegetation^16,17^, neo-formation of magnetic minerals^19,20^ and even bacterial action^27,28^. Because of providing simple, rapid, nondestructive and inexpensive measurements, soil magnetic properties have been used to delineate soil boundaries^29^, discriminate soil moisture conditions^30,31^, detect anthropogenic pollution^21,32^, as well as reconstruct paleoclimate^33,34^. Contrasting to the well-documented Fe dynamics during natural pedogenesis, a comprehensive understanding of the variations and controls of Fe oxides and magnetism changes during anthropedogenesis of paddy soils strongly affected by human activities is poorly constrained. The natural pedogenic controls on Fe and magnetism evolution may be superseded by human activities^35^ that alter the rate and trajectory of net Fe dynamics either directly (e.g., Fe additions or leaching by irrigation) or indirectly (e.g., Fe transformations by artificial flooding and draining).

Paddy soils make up the largest anthropogenic wetlands on earth and thus represent a key component of the Fe geochemical cycle at the Earth’s surface. Previous studies have investigated the changing status of Fe oxides and magnetic properties at a given stage of paddy soil development through a comparison with the initial parent material. For instance, paddy soils showed higher profile differentiation of Fe oxides^36–39^ and exhibited lower magnetic susceptibility^40–44^ than their well-drained counterparts in many regions of the world. However, little is known about the successive changes of different Fe pools and magnetic parameters during paddy soil evolution that is required to identify process rates and thresholds of Fe dynamics. In addition, paddy soils may originate from many types of soils in pedological terms or different parent materials. Yet, few studies have involved the effects of parent materials (or original soils) on the rates and trajectories of Fe and magnetism evolution in paddy soils.

Paddy soil chronosequence provides a valuable tool for investigating the phases and rates of property changes and the associated environmental thresholds^45^. In this study, we measured different forms of Fe oxides and magnetic parameters in two contrasting paddy soil chronosequences developed on calcareous marine sediment and acid Quaternary red clay in Southern China. Our objectives were to (i) investigate the dynamic changes in different Fe pools and magnetic properties during anthropedogenesis of paddy soils; (ii) identify the underlying mechanisms and processes controlling the phases and rates of Fe oxides and magnetism changes; and (iii) establish the influence of parent material and time span on Fe and magnetism evolution in paddy soils.

## Results

### Chronosequential changes in Fe concentrations

Total Fe concentration remained relatively constant in both uncultivated pedons, ranging from 28.22 to 30.34 g kg^−1^ in P0-MS derived from marine sediment and from 46.64 to 49.39 g kg^−1^ in P0-RC derived from Quaternary red clay (Table 1).The higher total Fe concentration in P0-RC than P0-MS was attributed to the differences in parent materials. Larger fluctuations of total Fe concentration were observed in all paddy soil profiles (Table 1), as demonstrated by the increasing standard deviations of total Fe concentration within 120 cm profile in both chronosequences (P0-MS, 0.92; P50-MS, 3.02; P300-MS, 4.78; P700-MS, 11.19; P1000-MS, 12.44; P0-RC, 1.37; P60-RC, 11.52; P150-RC, 1.40; P300-RC, 6.01). This variation occurred due to the relative enrichment of total Fe in the illuvial horizons as compared with the corresponding surface horizons (Table 1). The weighted-mean total Fe concentration within 120 cm profile increased across the calcareous paddy soil chronosequence developed on marine sediment (P0-MS, 29.50 g kg^−1^; P50-MS, 36.55 g kg^−1^; P300-MS, 37.69-MS g kg^−1^; P700, 39.72-MS g kg^−1^; P1000-MS, 42.51 g kg^−1^), but decreased across the acid paddy soil chronosequence developed on Quaternary red clay (P0-RC, 44.45 g kg^−1^; P60-RC, 40.38 g kg^−1^; P150-RC, 35.68 g kg^−1^; P300-RC, 36.62 g kg^−1^). Our results suggest that both accumulation and depletion of Fe could occur during paddy soil evolution depending on the characteristics of parent materials and landscape positions.

**Table 1:** Fe concentrations of the studied paddy soil chronosequences developed on calcareous marine sediment and acid Quaternary red clay.

Layer	Depth	Weakly bound Fe	Oxide bound Fe	Silicate bound Fe	Total Fe	Layer	Depth	Weakly bound Fe	Oxide bound Fe	Silicate bound Fe	Total Fe
	cm	g kg^−1^	g kg^−1^	g kg^−1^	g kg^−1^		cm	g kg^−1^	g kg^−1^	g kg^−1^	g kg^−1^
**P0-MS: Uncultivated soil (time zero) developed on marine sediment**						**P0-RC: uncultivated soil (time zero) developed on Quaternary red clay**					
C1	0–30	5.46 (18)a	4.10 (13)	20.79 (69)	30.34	A	0–12	1.68 (3)	32.54 (70)	12.42 (27)	46.64
C2	30–60	5.21 (17)	3.90 (13)	20.45 (70)	29.56	Br1	12–47	2.13 (4)	36.05 (73)	11.21 (23)	49.39
C3	60–90	4.93 (16)	2.60 (9)	22.40 (75)	29.92	Br2	47–87	2.08 (4)	35.31 (76)	9.27 (20)	46.66
C4	90–120	4.78 (17)	2.17 (8)	21.27 (75)	28.22	C	87–120	1.91 (4)	36.64 (76)	9.97 (21)	48.53
**P50-MS: 50-yr paddy soil developed on marine sediment**						**P60-RC: 60-yr paddy soil developed on Quaternary red clay**					
Ap1	0–16	3.35 (10)	5.94 (17)	25.11 (73)	34.40	Ap1	0–10	0.91 (5)	9.55 (55)	7.00 (40)	18.40
Ap2	16–25	1.01 (3)	8.33 (24)	24.76 (73)	34.11	Ap2	10–18	2.30 (7)	28.34 (81)	4.55 (13)	37.04
Bg1	25–50	2.09 (6)	6.69 (20)	24.32 (73)	33.11	Br1	18–30	4.12 (13)	17.98 (55)	10.65 (33)	35.29
Bg2	50–70	1.49 (4)	7.79 (21)	27.07 (74)	36.36	Br2	30–60	6.84 (15)	28.47 (61)	11.52 (25)	43.42
Bg3	70–100	0.88 (2)	9.95 (26)	27.58 (72)	38.41	Br3	60–85	5.68 (12)	32.72 (70)	8.23 (18)	41.63
BCg	100–120	1.09 (3)	10.63 (26)	29.32 (71)	41.04	BC	85–120	5.53 (12)	31.63 (70)	8.34 (18)	43.73
**P300-MS: 300-yr paddy soil developed on marine sediment**						**P150-RC: 150-yr paddy soil developed on Quaternary red clay**					
Ap1	0–17	2.72 (8)	5.85 (17)	24.90 (74)	33.47	Ap1	0–11	3.61 (10)	19.76 (56)	11.83 (34)	32.65
Ap2	17–26	0.84 (3)	8.35 (28)	20.81 (69)	30.00	Ap2	11–20	6.46 (17)	18.51 (49)	12.81 (34)	34.33
Bg1	26–43	1.02 (3)	10.75 (28)	25.98 (69)	37.75	Br1	20–28	3.45 (10)	21.76 (63)	9.59 (28)	36.40
Bg2	43–70	1.46 (4)	10.48 (25)	29.32 (71)	41.26	Br2	28–35	2.11 (6)	26.71 (75)	6.94 (19)	38.72
Bg3	70–90	2.62 (6)	9.41 (22)	30.51 (72)	42.53	Br3	35–48	2.39 (7)	25.73 (75)	6.39 (19)	36.64
BCg	90–120	2.16 (6)	7.91 (22)	24.93 (72)	35.00	BC	48–64	4.36 (12)	27.90 (77)	3.80 (11)	37.19
**P700-MS: 700-yr paddy soil developed on marine sediment**						C	64–120	3.43 (9)	27.44 (72)	7.19 (19)	35.23
Ap1	0–15	4.57 (2)	3.77 (13)	19.73 (75)	28.07	**P300-RC: 300-yr paddy soil developed on Quaternary red clay**					
Ap2	15–22	2.49 (9)	6.34 (22)	19.59 (69)	28.41	Ap1	0–10	3.91 (18)	13.87 (62)	4.53 (20)	24.87
Eg	22–42	1.20 (4)	8.89 (31)	18.99 (65)	29.08	Ap2	10–22	3.78 (15)	18.30 (73)	3.05 (12)	27.93
Btg1	42–60	0.79 (1)	24.38 (46)	27.72 (52)	52.89	Br1	22–45	2.23 (7)	25.73 (75)	6.29 (18)	43.26
Btg2	60–90	0.97 (2)	23.32 (46)	28.18 (52)	52.48	Br2	45–65	2.48 (7)	25.96 (78)	4.69 (14)	40.45
Ab	90–112	0.50 (2)	2.46 (7)	29.89 (91)	32.85	BC	65–120	2.86 (10)	19.67 (71)	5.24 (19)	35.96
Bb	112–120	0.45 (1)	7.88 (25)	23.48 (74)	31.81						
**P1000-MS: 1000-yr paddy soil developed on marine sediment**											
Ap1	0–16	3.60 (1)	5.57 (20)	18.94 (69)	28.11						
Ap2	16–25	0.51 (1)	13.17 (33)	26.11 (66)	39.79						
Btg1	25–50	1.14 (3)	15.39 (35)	27.63 (62)	44.16						
Btg2	50–70	3.27 (6)	19.02 (37)	29.68 (57)	51.98						
Btg3	70–85	3.55 (6)	25.68 (41)	34.06 (54)	63.29						
Ab	85–100	2.58 (9)	5.32 (18)	21.51 (73)	29.42						
Bb	100–120	2.56 (7)	12.29 (32)	23.25 (61)	38.09						

^a^Numbers in the parentheses represent the fraction of different Fe oxides to total Fe.

**Figure 1:**
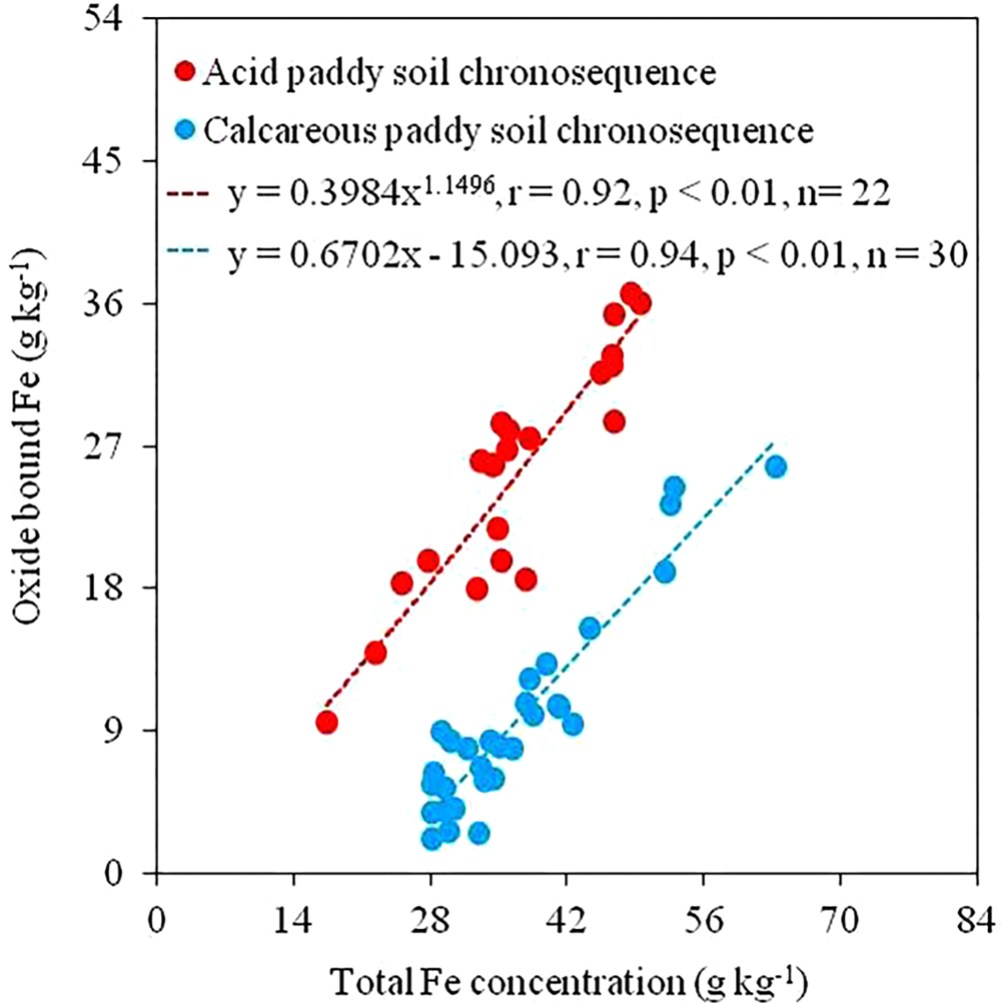
Relationship between the oxide bound Fe and total Fe in calcareous and acid paddy soil chronosequences.

Selective extractions showed that the contributions of different Fe pools to total Fe varied markedly between two paddy soil chronosequences developed on different parent materials (Table 1). Silicate bound Fe was the dominant Fe pool in the calcareous paddy soil chronosequence developed on marine sediment, which corresponded to 52~91% of the total Fe and fluctuated with both soil depth and increasing paddy cultivation age (Table 1). In contrast, oxide bound Fe was the major Fe pool in the acid paddy soil chronosequence developed on Quaternary red clay, which represented 49~81% of the total Fe and tended to increase with soil depth and fluctuated with increasing paddy cultivation history (Table 1). The weakly bound, poorly crystalline Fe pool was the smallest Fe pool in both chronosequences, contributing to 1~16% and 4~18% of the total Fe in the calcareous and acid paddy soil chronosequence, respectively (Table 1). There were strong correlations between the oxide bound Fe and total Fe in both chronosequences, with the correlation coefficient of 0.94 (*n* = 30, *p* < 0.01) and 0.92 (*n* = 22, *p* < 0.01) for the calcareous and acid paddy soil chronosequence (Fig. 1), respectively, suggesting that variations of Fe contents during paddy soil evolution were mainly caused by the oxide bound Fe.

### Chronosequential changes in magnetic parameters

**Figure 2:**
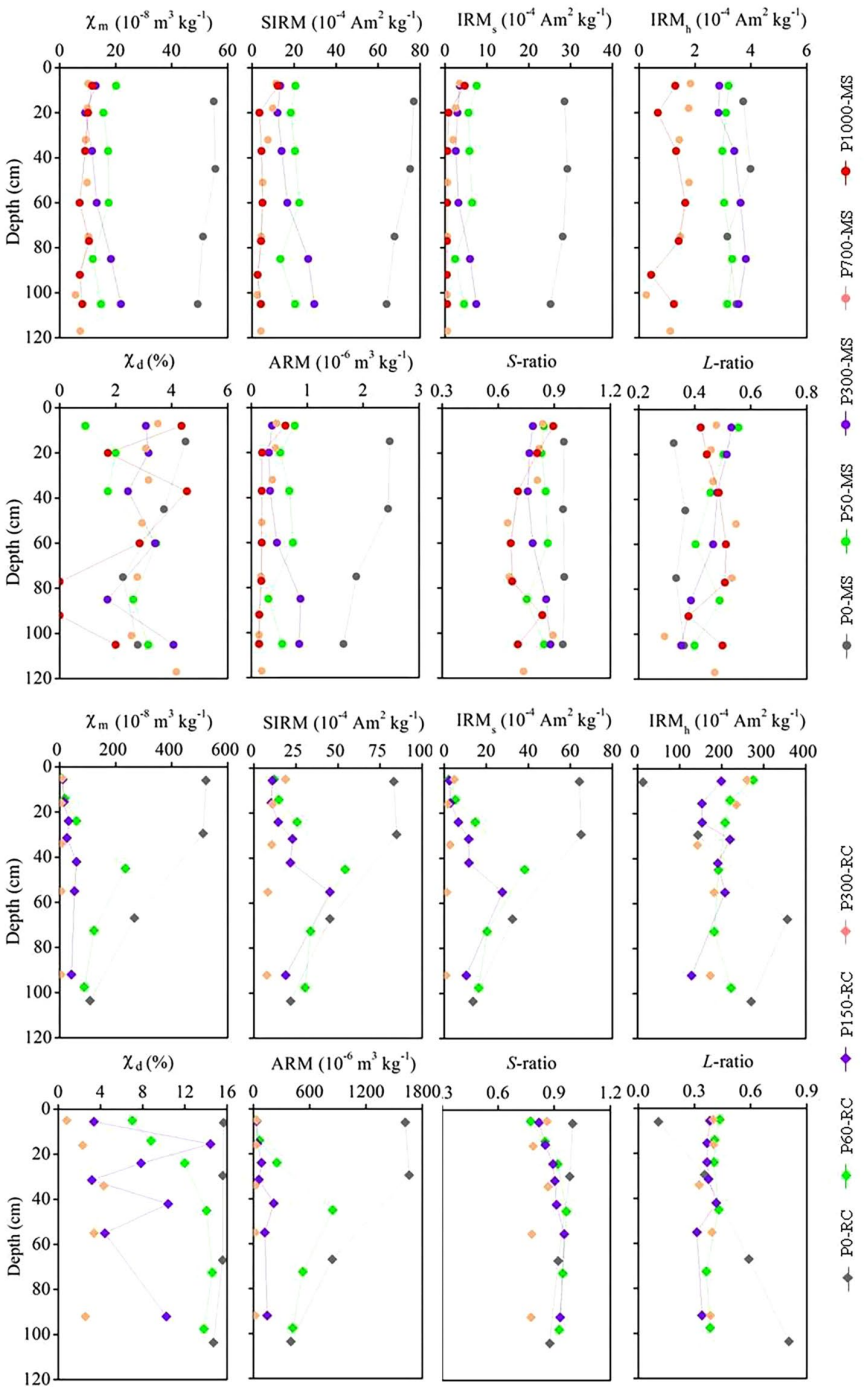
Changes in magnetic properties in the calcareous paddy soil chronosequence developed on marine sediment (P0-MS, P50-MS, P100-MS, P300-MS, P700-MS, and P1000-MS) and acid paddy soil chronosequence developed on Quaternary red clay (P0-RC, P60-RC, P150-RC, and P300-RC).

χ_m_, SIRM, IRM_s_, and ARM were much higher in the uncultivated soils (P0-MS, P0-RC) than the corresponding paddy soils, and these magnetic parameters decreased rapidly during the initial stage of paddy soil evolution (<100 years) and then declined gradually as paddy soils age (>100 years) in both chronosequences (Fig. 2). The consistent decrease of χ_m_, SIRM, IRM_s_, and ARM with paddy cultivation history irrespective of the different parent materials (Fig. 2) resulted in strong correlations between different magnetic parameters in both chronosequences (r > 0.90, p < 0.01, Table 2), and provided an opportunity to use these magnetic parameters for estimating the relative ages of paddy soils. Changes in the distribution of IRM_*h*_ with paddy cultivation age were, however, much different from that of MS, SIRM, IRM_s_, and ARM in both chronosequences (Fig. 2). The weighted-mean value of IRM_*h*_ within 120 cm profile in the calcareous paddy soil chronosequence developed on marine sediment decreased gradually from 3.58 × 10^−4^ A m^2^ kg^−1^ in the uncultivated pedon (P0-MS) to 3.14 × 10^−4^ A m^2^ kg^−1^ after 300 years of rice cultivation (P300-MS), and then declined rapidly to less than 1.30 × 10^−4^ A m^2^ kg^−1^ as paddy soils age (>700 years) (Fig. 2). IRM_*h*_ in the acid paddy soil chronosequence developed on Quaternary red clay exhibited opposite trend in the upper (<50 cm) and lower soil layers (>50 cm), which was respectively lower (<50 cm) and higher (>50 cm) in the uncultivated pedon (P0-RC) than that of paddy soils (Fig. 2). χ _d_ values showed variations in the vertical profiles in both calcareous and acid paddy soil chronosequence, and respectively fluctuated and decreased with paddy cultivation age (Fig. 2). *S*-ratio varied from 0.65 to 0.95 and from 0.77 to 1.00 in the calcareous and acid paddy soil chronosequence, respectively, which tended to decrease with paddy cultivation age in both chronosequences (Fig. 2). *L*-ratio varied from 0.29 to 0.56 and from 0.11 to 0.81 in the calcareous and acid paddy soil chronosequence, respectively, and there was no time-dependent changes for *L*-ratio in both chronosequences (Fig. 2).

**Table 2:** Correlations between different magnetic parameters in the calcareous and acid paddy soil chronosequences.

Calcareous paddy soil chronosequence					Acid paddy soil chronosequence				
	χ_m_	SIRM	IRM*s*	ARM		χ_m_	SIRM	IRM*s*	ARM
χ_m_	1				χ_m_	1			
SIRM	0.991				SIRM	0.951			
IRM*s*	0.995	0.990			IRM*s*	0.968	0.996		
ARM	0.979	0.986	0.980	1	ARM	0.995	0.948	0.964	1

The vertical distributions of χ_m_, SIRM, IRM_s_, IRM_*h*_, and ARM were relatively uniform in the calcareous paddy soil chronosequence developed on marine sediment, contrasting markedly with the larger profile fluctuations in the acid paddy soil chronosequence developed on Quaternary red clay (Fig. 2). The values of χ_m_, SIRM, IRM_s_, IRM_*h*_, and ARM in the calcareous paddy soil chronosequence were lower than those in the acid paddy soil chronosequence, and the discrepancies declined with paddy cultivation age (Fig. 2). Our results demonstrated the significant influence of parent material on paddy soil magnetism and this influence tended to diminish with time.

## Discussion

### Variations and controls of different Fe pools during paddy soil evolution

Our study using a chronosequence approach demonstrated that Fe was mobilized and translocated within profile during paddy soil evolution, which was confirmed by the increasing differentiation of Fe concentration and speciation within different selective extractions in both calcareous and acid paddy soil chronosequences (Table 1). Our results are consistent with prior observations that rice cultivation influences Fe differentiation within paddy soils, irrespective of different parent materials^36,39,44,46,47^. The alternating flooding and drying processes during rice cultivation are expected to cause changes in soil pH and Eh^48^, which would result in coupled reduction-oxidation and eluviation-illuviation processes of Fe in paddy soils^46,49^ and thus lead to the formation of diagnostic horizons and features (Table S1) characterizing Fe distribution and redistribution as paddy soils age. In addition to the artificial flooding and drainage, seasonal fluctuations of groundwater level could also induce changes in soil redox potential^50,51^ that favor Fe reduction and depletion in the lower horizons of paddy soils with shallow groundwater level (Table 1).

**Figure 3:**
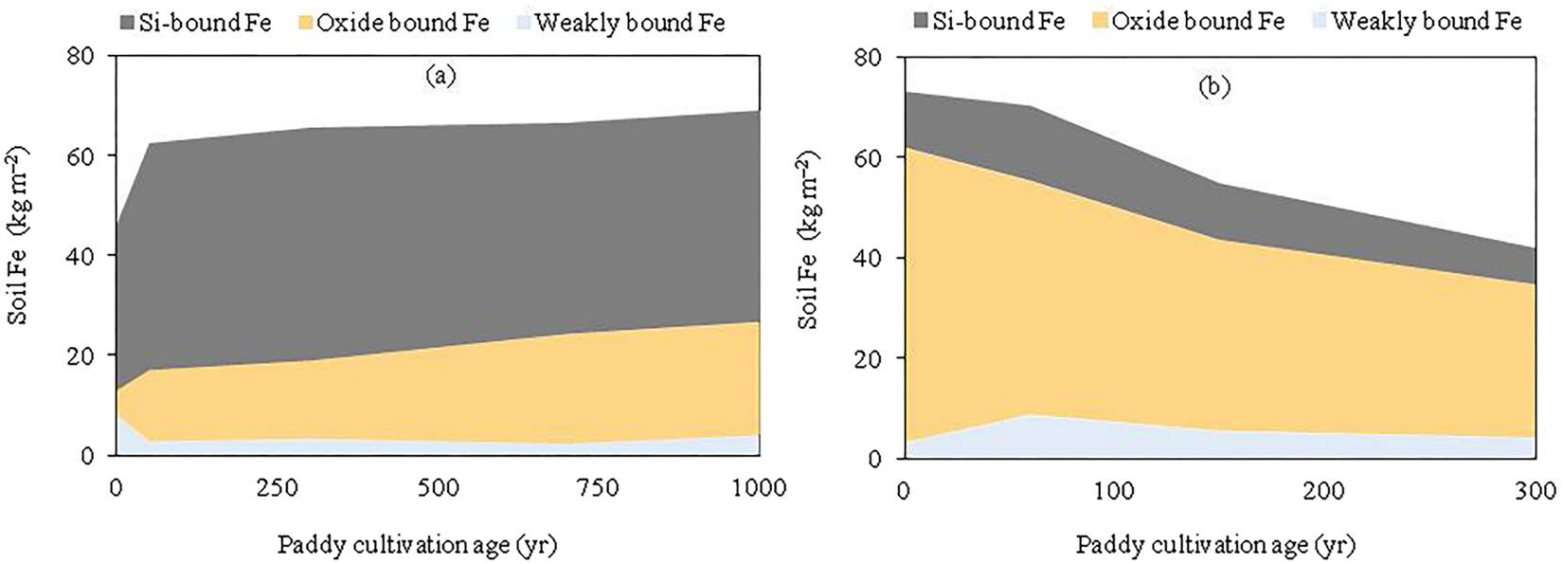
Changes in Fe mass within 0~120 cm soil layer during paddy soil development on calcareous marine sediment (**a**) and acid Quaternary red clay (**b**).

The comparison of calcareous and acid paddy soil chronosequences developed on marine sediment and Quaternary red clay showed significant influences of parent materials on the rates and trajectories of Fe evolution (Fig. 3). Total Fe and oxide bound Fe in the calcareous paddy soil chronosequence increased consistently from 47 and 5 kg m^−2^, respectively, in the uncultivated soil (P0-MS) to 69 and 23 kg m^−2^ after 1000 years of rice cultivation (P1000-MS) (Fig. 3). The average increasing rate of total Fe (0.32 kg m^−2^ yr^−1^) and oxide bound Fe (0.19 kg m^−2^ yr^−1^) during the first 50 years of rice cultivation was, respectively, 36- and 28-fold greater than that between 50- and 1000-yrs time period (Fig. 3). The silicate bound Fe in the calcareous paddy soil chronosequence increased gradually from 31 kg m^−2^ in the uncultivated soil (P0-MS) to 46 kg m^−2^ in the 50-yr paddy soil (P50-MS) and then remained relatively constant in the progressively older paddy soils (Fig. 3). The weakly bound Fe in the calcareous paddy soil chronosequence decreased at a rate of 0.12 kg m^−2^ yr^−1^ during the initial 50 years of rice cultivation while it showed minimal changes thereafter (Fig. 3). In a sharp contrast, total Fe and oxide bound Fe in the acid paddy soil chronosequence decreased consistently from 73 and 59 kg m^−2^, respectively, in the uncultivated soil (P0-RC) to 42 and 31 kg m^−2^ after 300 years of rice cultivation (P300-RC) (Fig. 3). The average decreasing rate of total Fe (0.04 kg m^−2^ yr^−1^) and oxide bound Fe (0.20 kg m^−2^ yr^−1^) during the first 60 years of rice cultivation was, respectively, 0.36- and 2-fold of that between 60- and 300-year time period (Fig. 3). The silicate bound Fe and weakly bound Fe increased initially from 11 and 3 kg m^−2^ in the uncultivated soil (P0-RC) to 15 and 9 kg m^−2^ in the 60-year paddy soil (P60-RC), and then declined gradually to 7 and 4 kg m^−2^ in the 300-year paddy soil (P300-RC) (Fig. 3).

Previous studies have shown that the critical redox potentials for Fe reduction and consequent dissolution are between + 300 mV and + 100 mV at pH 6~7, and −100 mV at pH 8,while at pH 5 appreciable Fe reduction occurs at + 300 mV^52^. The pH value of paddy soils derived from calcareous marine sediment and acid Quaternary red clay ranged from 6.3 to 8.6 and from 4.8 to 6.4, respectively (Table S2). The alkaline environment at the initial stage (0~50 years) of calcareous paddy soil evolution (Table S2) would impede loss of Fe from the profile, and thus an initial period of Fe accumulation was observed in the calcareous paddy soil chronosequence (Fig. 3). As pedogenesis proceeded and CaCO_3_ was gradually removed from the profile, the soil pH decreased (Table S2) and Fe accumulated at a lower rate in the later stages of calcareous paddy soil evolution (Fig. 3). In contrast, the acid environment (Table S2) together with the relatively high leaching potential in the acid paddy soil chronosequence are expected to promote Fe mobilization and leaching loss after artificial flooding (Fig. 3). This was confirmed by the rapid decrease of Fe and clay content in the acid paddy soil chronosequence in the sloping upland area, which contrasted markedly with the gradual increase of Fe and clay content in the calcareous paddy soil chronosequence in the plain area (Fig. 3, Table S2). In addition to the reductive leaching^38,44,46,47^, particle-facilitated leaching and transport of Fe may also explain the rapid decrease Fe of in the acid paddy soil chronosequence developed on the sloping upland area. Further studies on Fe transport by particles and colloids in terraced paddy soils at different slope positions are required to test this hypothesis.

### Phases and rates of magnetism changes during paddy soil evolution

**Figure 4:**
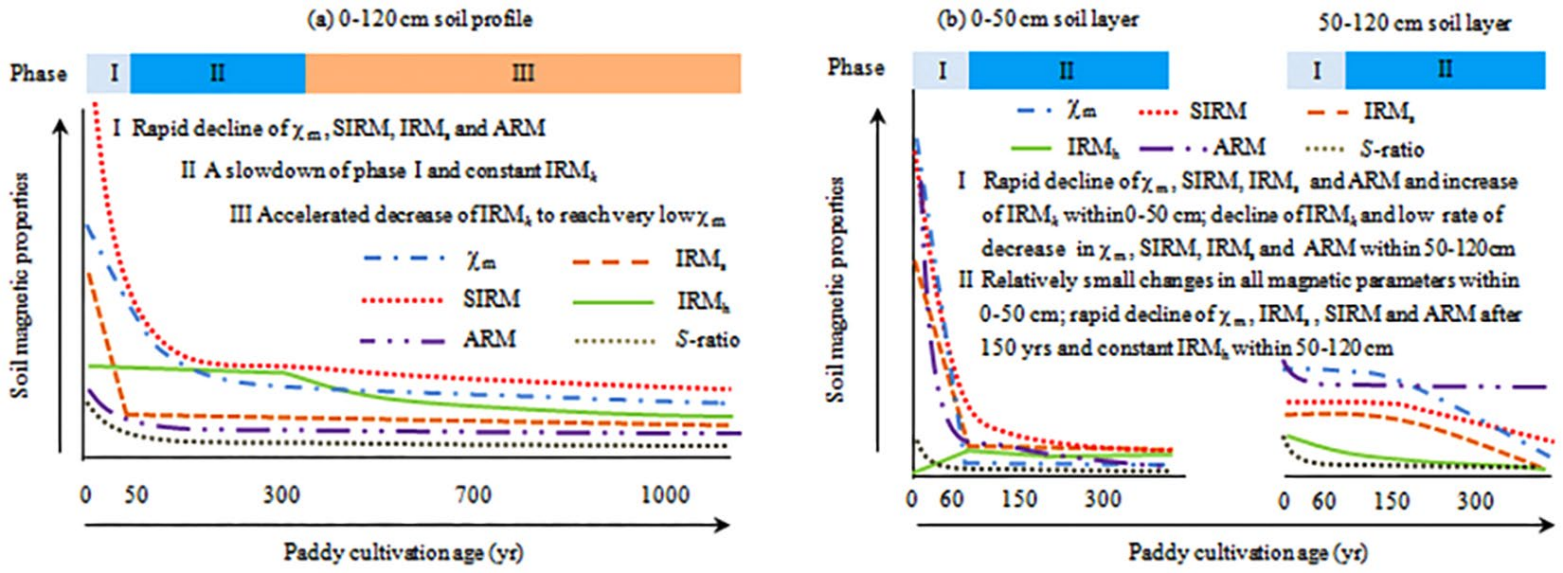
Phases and rates of magnetism changes during paddy soil development on calcareous marine sediment (**a**) and acid Quaternary red clay (**b**).

Previous studies have demonstrated that paddy soils exhibit lower magnetic susceptibility than their well-drained counterparts^40,42,44^, however, little is known about the rates of magnetism changes during long-term paddy soil evolution. Our study showed different phases and rates of magnetism changes during paddy soil development on calcareous marine sediment and acid Quaternary red clay (Fig. 4). The vertical distribution of magnetic parameters was uniform in the calcareous paddy soil chronosequence (Fig. 2) and we identified three periods/phases of magnetism changes based on the shifts in magnetic parameters (Fig. 4). The initial phase occurred within half a century and comprised rapid decreases in χ_m_, SIRM, IRM_*s*_, ARM and *S*-ratio, and a slow decline of IRM_*h*_ (Fig. 4). The weighted-mean value of χ_m_, SIRM, IRM_*s*_, ARM, *S*-ratio and IRM_*h*_ within the 120 cm profile in the 50-yr paddy soil (P50-MS) decreased by 78%, 73%, 80%, 72%, 26% and 12% respectively relative to the uncultivated soil (P0-MS). According to the physical meanings of the different magnetic parameters (Table S3), these changes suggest a rapid destruction of fine-grained maghemite and/or ultrafine magnetite during the initial stage of paddy soil evolution (0~50 years). The second phase lasted several centuries (50~300 years) comprising a relatively constant IRM_*h*_ and a slow rate of decline in χ_m_, SIRM, IRM_*s*_, ARM and *S*-ratio (Fig. 4). The rate of decrease in χ_m_, SIRM, IRM_*s*_, ARM and *S*-ratio within 120 cm profile at this stage (50~300 years) was less than 5% of that in the initial stage (0~50 years). These results suggest that ferrimagnetic minerals (magnetite and maghemite) decrease successively while the antiferromagnetic minerals (hematite and goethite) remain relatively constant within 50~300 years. In the third phase (700~1000 years), χ_m_, SIRM, IRM_*s*_, ARM and *S*-ratio showed minimal changes while IRM_*h*_ declined rapidly (Fig. 4), suggesting significant depletion of antiferromagnetic minerals (hematite and goethite) occurred after 700 years of paddy cultivation. This resulted in the lowest content of magnetic minerals in the oldest paddy soil (Figs 2 and 4). The rapid decline of IRM_*h*_ after 700 years coincided with the complete removal of CaCO_3_ (Table S2). Higher soil pH due to the existence of CaCO_3_ has been confirmed to retard the transformation of silicate Fe to secondary Fe oxides as well as the reduction and leaching loss of Fe oxides^4^. We thus hypothesize that the complete removal of CaCO_3_ after 700 years of paddy cultivation would promote the reduction and leaching loss of Fe oxides (including the antiferromagnetic minerals) and consequently result in the rapid decrease of IRM_*h*_. Further work needs to be done to establish the link between CaCO_3_ content and the formation and transformation of magnetic minerals.

The acid paddy soil chronosequence showed two phases of magnetism changes, but the changes in the 0–50 cm soil layer were completely different from that in the 50–120 cm soil layer. In the first phase (0~60 years), χ_m_, SIRM, IRM_*s*_, and ARM declined but IRM_*h*_ increased rapidly in the 0–50 cm soil layer, while all these magnetic parameters declined in the 50–120 cm soil layer (Fig. 4). The weighted-mean value of χ_m_, SIRM, IRM_*s*_, and ARM within 0–50 cm in the 60-yr paddy soil (P60-RC) decreased by 98%, 86%, 94%, and 82% respectively relative to the uncultivated soil (P0-RC). In the second phase (60~300 years), there were minimal changes of different magnetic parameters (χ_m_, SIRM, IRM_*s*_, IRM_*h*_ and ARM) in the 0–50 cm soil layer, while χ_m_, SIRM, IRM_*s*_, and ARM decreased rapidly after 150 years of paddy cultivation in the 50–150 cm soil layer (Fig. 4). Previous studies have demonstrated the relations between Fe oxides, soil color and soil formation^4^. Hematite-containing soils (usually with associated goethite) have hues between 5YR and 10R, whereas goethite-containing soils with no hematite have hues between 7.5YR and 2.5Y. Soils with lepidocrocite and ferrihydrite covered the hues in-between-range of 5YR~7.5YR with values > 6 for lepidocrocite and <6 for ferrihydrite. Based on soil color (Table S1) and the magnetic properties (Fig. 2), the magnetic minerals were dominated by goethite within 0–50 cm and by hematite within 50–120 cm after 300 years of paddy cultivation.

The evident depletion of magnetism during the anthropedogenesis of paddy soils (Figs 2 and 4) contrasts markedly with the observations of magnetic enhancement during natural pedogenesis^17–25^. The increase of magnetic susceptibility during natural soil formation under predominantly oxic weathering conditions has been attributed to the formation of nano-sized magnetite and/or maghaemite, irrespective of the different parent materials^20,21,24^. The periodic submergence and drainage in paddy soils alters this trajectory of magnetism changes by destroying the ferrimagnetic minerals. Previous studies have shown that reducing conditions in paddy soils enhance the dissolution of ferrimagnetic minerals, leading to reduced magnetic properties in paddy soils relative to their well-drained counterparts^40,42,44^. Our results also showed that magnetic parameters (χ_m_, SIRM, IRM_*s*_, ARM and *S*-ratio) declined rapidly during the early stage of paddy soil development on different parent materials (Fig. 4). The overall magnetic depletion during anthropedogenesis of paddy soils over a millennium time scale (Fig. 4) provides an opportunity to use magnetic susceptibility for estimating the relative age of paddy soils. In addition, our study using a chronosequence approach demonstrated that the parent material and time-span influence the rates of magnetic depletion in different phases of magnetic property development (Fig. 4).

### Conclusion remarks on the parent material effects

Paddy soil evolution under the influence of artificial submergence and drainage caused changes in soil moisture regimes and redox conditions with both time and depth that controlled Fe transport and redistribution, leading to increasing profile differentiation of Fe oxides (total Fe, oxide-bound Fe, silicate-bound Fe and weakly-bound Fe), rapid decrease of magnetic parameters (e.g., χ_m_, SIRM, IRM_s_, and ARM), and formation of diagnostic horizons (i.e., anthraquic epipedon and hydragric horizon) and features (i.e., anthraquic moisture regime), irrespective of the different parent materials. However, a comparison of the two contrasting paddy soil chronosequences developed on calcareous marine sediment and acid Quaternary red clay demonstrated significant influence of initial parent material characteristics (e.g., pH, Fe content and compositions, weathering degree and landscape positions) on the rates and trajectories of Fe oxides evolution as well as on the phases and rates of magnetism changes, but this influence diminished with time as prolonged rice cultivation drove paddy soil evolving to common pedogenic features. Yet, it remains to be evaluated whether the initial parent material affects the rate of chemical convergence or how long it takes for the parent material effects to be nullified. This is because some of the properties of parent materials persist following long-term paddy soil management over a millennial time scale^53,54^, which is known as the pedological memory and inheritance. Paddy soils may originate from many types of soils in pedological terms showing considerable differences in weathering degree and their initial constitutions^45^. We suggest extensively investigate iron and magnetism changes in paddy soil chronosequences developed on different parent materials and establish the linkage between the expected different evolutional patterns. This will not only provide basic data that are necessary to develop quantitative models of Fe and magnetism changes, but also offers an opportunity to use the established models for predicting the future evolution trends.

## Materials and Methods

### Study area and sampling sites

The studied paddy soil chronosequences were located respectively on a coastal plain in Cixi County, Zhejiang Province (between 121°2′–121°36′ E and 30°2′–30°19′ N) and on a slope upland in Jinxian County, Jiangxi Province (116°1′–116°32′ E and 28°2′–28°26′ N), in Southern China (Fig. S1). Cixi county has a mean annual air temperature of 16 °C, with yearly extremes ranging from −5 °C to 37 °C, and a mean annual precipitation of 1325 mm of which 73% is concentrated in the rice paddy flooding season (i.e., April to October). The coastal plain ranges from 2.6 m to 5.7 m above sea level, and slopes gently towards the northeast (Fig. S1). Soils in the studied area were developed on calcareous marine sediment from the East China Sea, which received large amounts of terrigenous materials from the nearby Qiantang and Yangtze Rivers^53^. Step-by-step land reclamation of the tidal mudflat through successive dyke building^55^ has resulted in a chronosequence with different stages of soil development^53^. Rice (*Oryza sativa* L.) cultivation in the lower areas where fresh water is readily available for irrigation generally began after five years of dyke building when the salt concentration decreased to agronomically tolerable levels. Sites with 50, 300, 700 and 1000 years of rice cultivation history (i.e., P50-MS, P300-MS, P700-MS and P1000-MS) were identified (Fig. S1) based on the chronology of dyke construction^55^. In addition, an uncultivated mud beach profile (P0-MS) was selected to represent the original soil (parent material, time zero) of the paddy soils (Fig. S1). Parent material homogeneity in the inter- and intra-profiles of the studied chronosequence (P0-MS, P50-MS, P300-MS, P700-MS and P1000-MS) has been evaluated by making use of various soil attribute parameters^56,57^. Details of these profiles (P0-MS, P50-MS, P300-MS, P700-MS and P1000-MS) and the soil chronosequence recognization have been given by Chen *et al*.^53,57^ and Huang *et al*.^58,59^. Jinxian County has a mean annual air temperature of 17 °C, with yearly extremes from 5 °C to 40 °C, and a mean annual precipitation of 1587 mm, of which 79% is concentrated in the rice paddy flooding season (i.e., April to October). Terraced paddy fields are a common feature in this area. Soils at the bottom of slopes were generally the first to be converted to paddy field; as population pressure increased, lands upslope were progressively brought into paddy cultivation. Thus, these hillside terraces, with increasing cultivation age from the top to the bottom of the slopes, provide soil chronosequences. Soils were derived primarily from acid Quaternary red clays, which were highly weathered, clay rich and phosphorus deficient^60,61^. A paddy soil chronosequence (Fig. S1) consisting of three profiles with approximately 60 (P60-RC), 150 (P150-RC), and 300 years (P300-RC) of paddy cultivation history, and an uncultivated natural soil profile (P0-RC) representing the original soil (parent material, time zero) of the paddy soils were identified. The history of paddy cultivation in the older profile at the bottom of the slope was determined from local historical literature from when the nearby villages were settled. For the newer profile at the top of the slope, information was obtained by questioning the local farmers. The uncultivated soil profile at the highest slope position was treated as the original soil, i.e., time zero with respect to paddy cultivation. The relative ages of the collected paddy soils were confirmed by Han (2012)^60^ using the profile development index (PDI) according to Harden (1982)^62^. Details of these profiles (P0-RC, P60-RC, P150-RC, and P300-RC) were given by Han^60^, Han *et al*.^61^, and Huang *et al*.^58,59^, who investigated pedogenic changes in basic soil properties, clay minerals and phosphorus fractions. The studied chronosequence was also a toposequence, which complicated the interpretation of the results^49^. In general, time of cultivation had the more significant role, since soil moisture regimes of paddy soils were maintained similarly. In hilly regions, terrace construction greatly reduced water loss and soil erosion and substantially weakened the influence of topography on pedogenesis. Additionally, all four sites were on geomorphically stable topographic positions with low slope gradient (<5°), minimizing the effect of local erosion and deposition. Thus, the role of topography was not separately analyzed for different sampling sites, as we ascribed differential pedogenesis to the difference in time available for pedogenesis.

### Soil sampling and description

Within each area of identical paddy cultivation history, one representative profile was chosen for soil sampling based on soil landscape and geomorphological characteristics of that area. All soil samples were collected when the fields were drained after rice harvest. Soil profiles were described and sampled according to genetic horizons following standard field description guidelines^63,64^. The uncultivated soil profiles (P0-MS, P0-RC) in both chronosequences were generally homogeneous throughout its depth, with no visually discernible horizon differentiation (Fig. S2). In contrast, the paddy soil profiles showed complicated patterns with depth due to anthropedogenesis and consisted of an anthrostagnic epipedon, including the cultivated horizon (Ap1) and the plow pan (Ap2), and a hydragric horizon (Br or Bg) (Fig. S2, Table S1). Differences in morphological properties, including soil color, texture and redoximorphic features, were also evident between the relatively younger pedon and the older ones in both chronosequences (Fig. S2, Table S1). The original soils of the two paddy soil chronosequences were defined as Primosols (P0-MS) and Ferrosols (P0-RC), respectively. The paddy soils were defined as Hapi-Stagnic Anthrosols (P50-MS, P300-MS, P60-RC, P150-RC), Fe-leachi-Stagnic Anthrosols (P700-MS, P300-RC), and Fe-accumuli-Stagnic Anthrosol (P1000-MS) by referring to Chinese Soil Taxonomy^65^ (Table S1). The detailed field descriptions and classifications of the soil profiles are given in Table S1.

### Analysis of basic soil physicochemical properties

After collection, samples of each soil horizon were dried at room temperature and then gently crushed using a wooden pestle and mortar and passed through a 2-mm nylon sieve. Soil bulk density was measured on the 100 cm^−3^ undisturbed soil cores by drying the cores for 24 h at 105 °C. The particle size distribution was determined by the pipette method and the clay content was defined as the mass percentage of particles <2 μm in diameter for the whole soil. Soil pH was determined at a 1:2.5 soil/solution ratio using distilled water and the carbonate content was determined using a Dietrich Fruhling pressure calcimeter according to the Institute of Soil Science, Chinese Academy of Sciences (1978)^66^. Soil organic carbon (SOC) was measured by the Walkley-Black wet oxidation method^67^ using the 149-μm fraction. Total nitrogen (N_tot_) was measured by Kjeldahl method^68^ and total phosphorus (P_tot_) was determined by HClO_4_-HF digestion followed by colorimetric analysis^66^. For total elemental analysis, soil samples (<74 μm) were fused by a mixture of 1:1 lithium metaborate and lithium tetraborate for 30 min in a 1000 °C muffle furnace and then were dissolved in 10% HNO_3_ + 1% HF solution. Total elemental concentrations including K, Na, Ca, Mg, Fe, Mn, Al, Si, Ti, and Zr were determined by inductively coupled plasma-optical emission spectrometry. We estimated the precision as 5~10% relative standard deviation based on replicates and standard samples (Geochemical Standard Reference Sample Soil, GSS-3). The measured data are listed in Table S2. The dynamic changes in basic soil physicochemical properties have been reported by Chen *et al*.^53^, Han *et al*.^61^, and Huang *et al*.^58,59^. Briefly, the calcareous paddy soil chronosequence developed on marine sediment over a millennium time scale showed three phases of pedogenesis: an initial phase during the first few decades (0~50 years) dominated by rapid desalinization, accumulation of topsoil organic matter and formation of a compacted plow pan (Table S1 and S2); the second phase lasted several centuries (50~700 years) comprising Fe and clay enrichment in the illuvial horizon, and the loss of phosphorus and Mn coincident with the near complete removal of CaCO_3_ (Table S2); in the third phase (>700 years), (trans-)formation and redistribution of metal oxides were accompanied by clearly visible hydromorphic patterns in paddy subsoils (Table S1, Fig. S2). The acid paddy soil chronosequence developed on Quaternary red clay over a centurial time scale showed rapid accumulation of SOC and increase of pH in surface paddy soils, loss of clay and Fe oxides with prolonged cultivation history (Table S1, Fig. S2), and shifts in phosphorus abundance and speciation as well as clay mineral compositions with both time and depth^58,61^.

### Extraction of Fe oxides and measurement of magnetic properties

Bulk soil samples were subjected to reducing agents with increasing strength to selectively extract major pools of Fe: (1) the Tamm’s extraction^69^; and (2) the citrate-bicarbonate-dithionite (CBD) extraction^70^. The Tamm’s extraction is a mixture of oxalic acid and ammonium oxalate, which was performed by shaking the sample-solution mixture in the dark over 4 h at 20 °C with a solid/liquid ratio of 1.25 g/50 ml. The Tamm’s method targets the extraction of weakly bound, short-range-ordered (SRO) and organic bound Fe^71^. For the extraction by CBD, soil samples were exposed to the reactant mixture at 80 °C for 30 min with a solid/liquid ratio of 0.5 g/25 ml. The CBD method extracts Fe in oxides and hydroxides (e.g., hematite, goethite, lepidocrocite) of all crystallinities—SRO and bulk crystalline^70^. In addition to the partial extractions, total Fe was dissolved in a HF-HClO_4_ mixture after calcination of soil organic matter at 450 °C. Fe concentrations in the extracted solutions were analyzed using an Inductively Coupled Plasma-Atomic Emission Spectrometer (ICP-AES, LAS Arras). We calculated the oxide bound Fe concentration by subtracting oxalate-extractable Fe from the CBD-extractable Fe and the silicate bound Fe was calculated by subtracting CBD-extractable Fe from the total Fe concentration. Fe mass (kg m^−2^) in the soil pedon (0–120 cm) was calculated by multiplying Fe concentrations by bulk density and thickness of soil horizons using the following equation:

1 $${{\rm{Fe}}}_{mass}=\sum _{i}^{n}{C}_{Fe}{D}_{i}{E}_{i}/100$$

where *C*_*Fe*_, *D*_i_, and *E*_i_ is, respectively, the Fe concentration (g kg^−1^), bulk density (g cm^−3^) and depth (cm) in the *i* horizon.

Magnetic parameters were measured and calculated according to Evans and Heller (2003)^21^ and Lu (2003)^42^. Briefly, magnetic susceptibility (χ_m_) was measured with a Bartington MS2 meter (Bartlington Instruments Ltd., Oxford, UK) at both low (0.47 kHz, χ _lf_) and high frequencies (4.7 kHz, χ _hf_). Frequency-dependent magnetic susceptibility (χ_d_) was calculated as [(χ_lf_ − χ_hf_)/χ_lf_] × 100%. Isothermal remanent magnetization (IRM) was produced in progressively increasing magnetic fields (i.e., 20 mT, 30 mT, 50 mT, 100 mT, 300 mT, 1000 mT) and then was determined under reverse magnetic fields (−300 mT, −100 mT, −20 mT) using a Molspin pulse magnetizer (Molspin Ltd., Newcastle on Tyne, UK). The induced remanence after imposing each magnetic field was measured in a Molspin spinner magnetometer. The anhysteretic remanent magnetization (ARM) was acquired in a steady field of 0.04 mT imposed on an AC field with decreasing amplitude from a maximum of 100 mT to 0 mT^33^. The IRM at 1000 mT was defined as saturation isothermal remanent magnetization (SIRM) and was used to calculate the soft isothermal remanent magnetization (IRM_s_), hard isothermal remanent magnetization (IRM_h_), *S*-ratio and *L*-ratio using the following formulas:

2 $${{\rm{IRM}}}_{s}=({\rm{SIRM}}-{{\rm{IRM}}}_{-\mathrm{20mT}})/2$$

3 $${{\rm{IRM}}}_{h}=({\rm{SIRM}}+{{\rm{IRM}}}_{-300{\rm{mT}}})/2$$

4 $$S-\mathrm{ratio}=[({\rm{SIRM}}-{{\rm{IRM}}}_{-300{\rm{mT}}})/{\rm{SIRM}}]/2$$

5 $$L-\mathrm{ratio}={{\rm{IRM}}}_{h}/[({\rm{SIRM}}+{{\rm{IRM}}}_{-100{\rm{mT}}})/2]$$

Detailed information and interpretations of the operationally defined Fe pools and measured magnetic parameters are given in Table S3.

### Data availability

All data generated or analyzed during this study are included in the article and attached in the Supplementary Information files.

## Electronic supplementary material

Supplementary data
