# Supplementary material for: Phases and rates of iron and magnetism changes during paddy soil development on calcareous marine sediment and acid Quaternary red-clay
Source: Sci Rep. 2018 Jan 11;8:444. doi: 10.1038/s41598-017-18963-x (PMC5765014; doi:10.1038/s41598-017-18963-x)
Supplement: Supplementary file 1 — Supplementary data [file 41598_2017_18963_MOESM1_ESM.pdf]

Phases and rates of iron and magnetism changes during paddy soil development on calcareous marine sediment and acid Quaternary red-clay

Laiming Huang[1, 2, 3, 4], Xiaoxu Jia[1, 3, 4], Ming'an Shao[1, 3, 4*], Liumei Chen[5], Guangzhong Han[6], Ganlin Zhang[2, 3, *]

[1] Key Laboratory of Ecosystem Network Observation and Modeling, Institute of Geographic Sciences and Natural Resources Research, Chinese Academy of Sciences, Beijing 100101, China

[2] State Key Laboratory of Soil and Sustainable Agriculture, Institute of Soil Science, Chinese Academy of Sciences, Nanjing 210008, China

[3] College of Resources and Environment, University of Chinese Academy of Sciences, Beijing 100049, China

[4] State Key Laboratory of Soil Erosion and Dryland Farming on the Loess Plateau, Institute of Soil and Water Conservation, CAS & MWR, College of Natural Resources and Environment, Northwest A & F University

[5] College of Resources and Environment, Zunyi Normal College, Zunyi 563002, China

[6] College of Resources and Environmental Sciences, Neijiang Normal College, Neijiang 641112, China

*Correspondence and requests for materials should be addressed to M. S. and G. Z. (shaoma@igsnrr.ac.cn and glzhang@issas.ac.cn)

Supplementary Tables

Table S1 Description and classification of the studied soil profiles.

Table S2 Basic soil physico-chemical properties of the studied profiles.

Table S3 Interpretations of the operationally defined Fe pools and measured magnetic parameters.

Supplementary Figures:

Fig. S1. Location of the study area and sampling sites of the two paddy soil chronosequences. Note: P0-MS, P50-MS, P100-MS, P300-MS, P700-MS, and P1000-MS are representative soil profiles with 0, 50, 100, 300, 700 and 1000 years of rice cultivation history developed on calcareous marine sediments in Cixi County, Zhejiang Province, China; P0-RC, P60-RC, P150-RC, and P300-RC are representative soil profiles with 0, 60, 150, and 300 years of rice cultivation history developed on acid Quaternary red-clay in Jinxian County, Jiangxi Province, China.

Fig. S2. The studied pedons of the two paddy soil chronosequences developed on calcareous marine sediment (P0-MS, P50-MS, P100-MS, P300-MS, P700-MS, and P1000-MS) in Cixi County, Zhejiang Province and acid Quaternary red-clay (P0-RC, P60-RC, P150-RC, and P300-RC) in Jinxian County, Jiangxi Province.

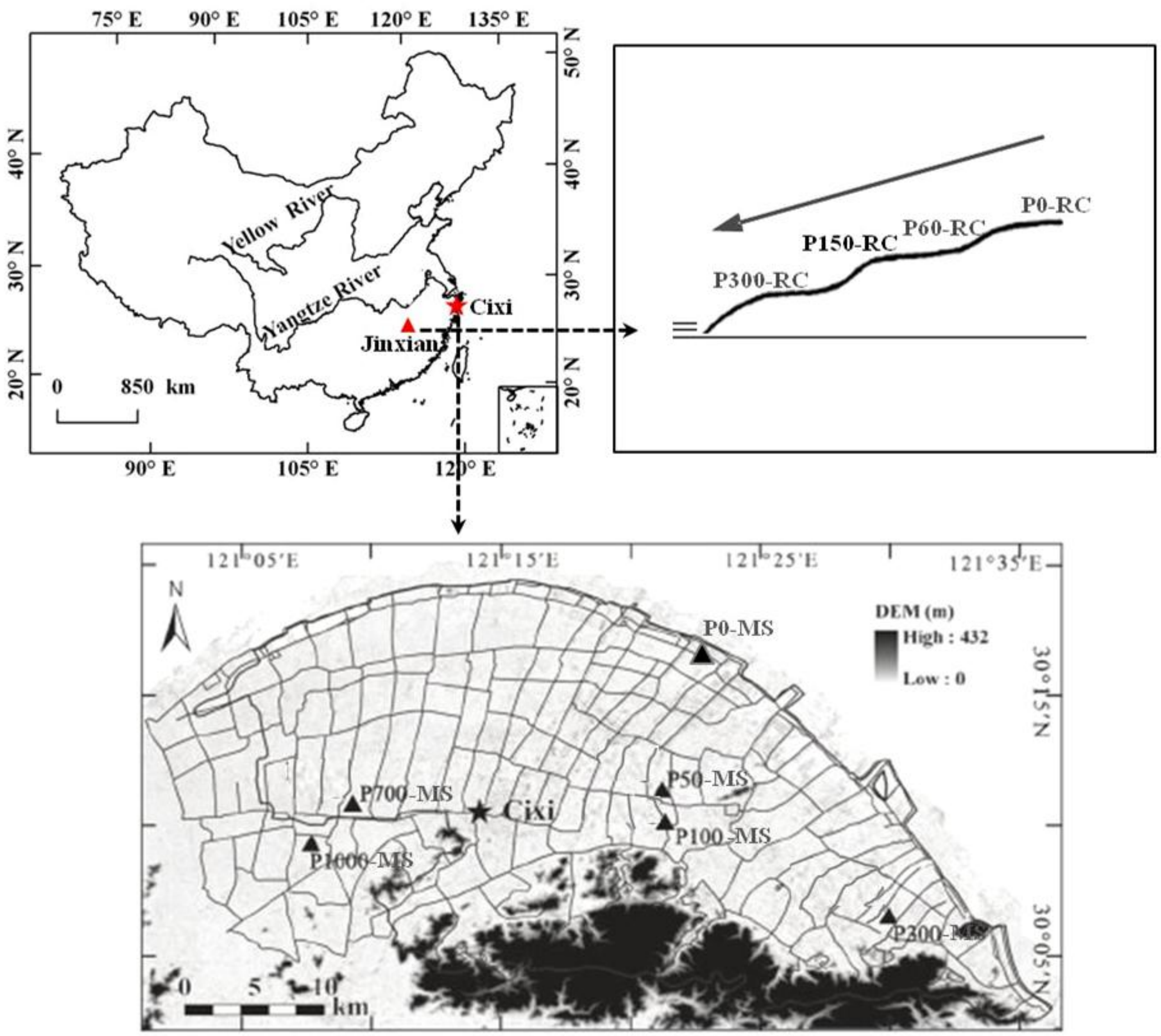

Figure S1. Location of the study area and sampling sites of the two paddy soil chronosequences. Note: P0-MS, P50-MS, P100-MS, P300-MS, P700-MS, and P1000-MS are representative soil profiles with 0, 50, 100, 300, 700 and 1000 years of rice cultivation history developed on calcareous marine sediments in Cixi County, Zhejiang Province, China; P0-RC, P60-RC, P150-RC, and P300-RC are representative soil profiles with 0, 60, 150, and 300 years of rice cultivation history developed on acid Quaternary red-clay in Jinxian County, Jiangxi Province, China. This map is generated by ArcGIS 9.0 (http://appsforms.esri.com/products/download/).

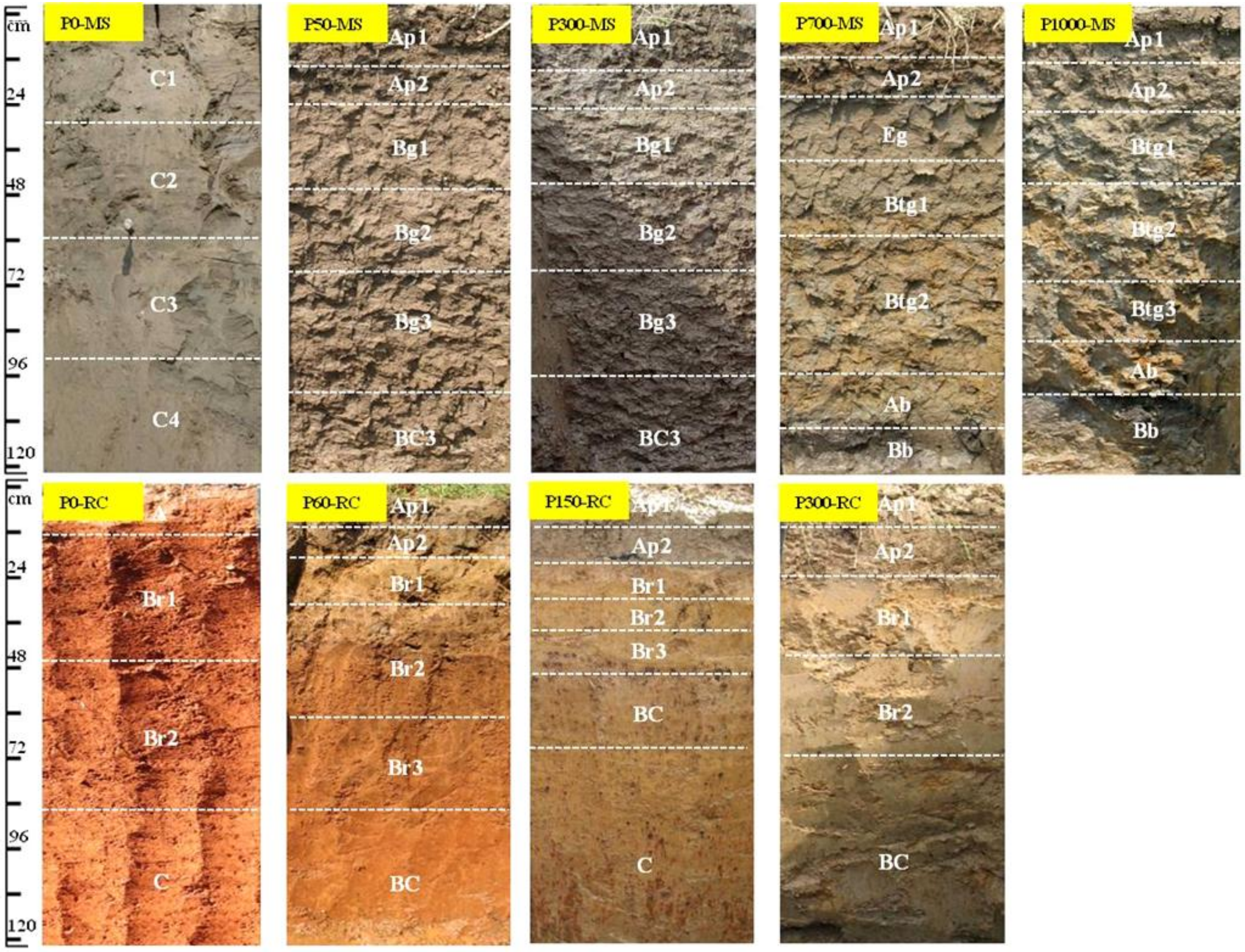

Fig. S2. The studied pedons of the two paddy soil chronosequences developed on calcareous marine sediment (P0-MS, P50-MS, P100-MS, P300-MS, P700-MS, and P1000-MS) in Cixi County, Zhejiang Province and acid Quaternary red-clay (P0-RC, P60-RC, P150-RC, and P300-RC) in Jinxian County, Jiangxi Province. Photos of P0-MS, P50-MS, P100-MS, P300-MS, P700-MS and P1000-MS were taken by Liumei Chen, and photos of P0-RC, P60-RC, P150-RC and P300-RC were taken by Guangzhong Han.

Horizon	Depth (cm)	Boundary [a]	Munsell color (dry)	Redoximorphic features [b]		Texture [c]	Structure [d]	Consistence (moist) [e]	Roots [f]
				Soft masses	Depletions				
**P0-MS: Uncultivated soil (time zero); classification [g]: Fluvisol (WRB), Entisol (ST), Primosol (CST)**									
C1	0–30	–	10YR 5/1	–	–	sil	ma	lo	–
C2	30–60	–	10YR 5/1	–	–	sil	ma	lo	–
C3	60–90	–	10YR 5/1	–	–	sil	ma	lo	–
C4	90–120	–	10YR 5/1	–	–	sil	ma	lo	–
**P50-MS: 50-yr paddy soil; classification: Hydragric Anthrosol (WRB), Typic Haplanthrept (ST), Hapi-Stagnic Anthrosol (CST)**									
Ap1	0–16	cs	10YR 5/2	f, 1, p, 2.5YR 5/8	–	sil	1, f, gr	lo	3f
Ap2	16–25	cs	10YR 5/4	–	–	sil	cdy	fi	1f
Bg1	25–50	gs	10YR 5/4	f, 1, p, 10YR 6/6	–	sil	1, f, sbk	fr	–
Bg2	50–70	gs	10YR 6/3	f, 1, p, 10YR 6/6	–	sil	1, f, sbk	fr	–
Bg3	70–100	gs	10YR 5/2	f, 1, p, 10YR 6/6	–	sil	1, f, sbk	fr	–
BCg	100–120	–	10YR 6/3	f, 1, p, 10YR 6/6	–	sil	1, f, sbk	fr	–
**P300-MS: 300-yr paddy soil; classification: Hydragric Anthrosol (WRB), Typic Haplanthrept (ST), Hapi-Stagnic Anthrosol (CST)**									
Ap1	0–17	cs	10YR 4/2	f, 1, p, 2.5YR 5/8	–	sil	1, f, gr	lo	3f
Ap2	17–26	cs	10YR 5/2	–	–	sil	cdy	fi	1f
Bg1	26–43	gs	10YR 5/3	c, 1, p, 10YR 6/6	–	sil	1, f, sbk	fr	–
Bg2	43–70	gs	10YR 4/4	c, 1, p, 10YR 6/6	–	sicl	1, f, sbk	fr	–
Bg3	70–90	gs	10YR 4/6	c, 1, p, 10YR 6/6	–	sicl	1, f, sbk	fr	–
BCg	90–120	–	10YR 5/4	c, 1, d, 10YR 6/6	–	sicl	1, f, sbk	fr	–
**P700-MS: 700-yr paddy soil; classification: Hydragric Anthrosol (WRB), Anthraquic Hapludalf (ST), Fe-leachi-Stagnic Anthrosol (CST)**									
Ap1	0–15	cs	10YR 4/2	c, 1, p, 2.5YR 5/8	–	sil	2, m, gr	lo	3f
Ap2	15–22	cs	5Y 5/1	–	c, 2, f, 5Y 5/1	sil	cdy	fi	1f
Eg	22–42	cs	2.5Y 5/2	–	c, 2, f, 5Y 5/1	sil	2, m, sbk	fr	–
Btg1	42–60	gs	2.5Y 5/2	m, 3, p, 10YR 6/6	c, 2, d, 2.5Y 5/1	sicl	3, c, sbk	fi	–
Btg2	60–90	as	2.5Y 5/2	m, 3, p, 10YR 6/6	c, 2, d, 2.5Y 5/1	sicl	3, c, sbk	fi	–
Ab	90–112	as	10YR 2/1	–	–	sicl	2, m, sbk	fi	–
Bb	112–120	–	5Y 5/1	m, 3, d, 10YR 6/6	–	sicl	3, c, sbk	fr	–
**P1000-MS: 1000-yr paddy soil; classification: Hydragric Anthrosol (WRB), Anthraquic Hapludalf (ST), Fe-accumuli-Stagnic Anthrosol (CST)**									
Ap1	0–16	cs	10YR 4/1	c, 1, p, 10YR 6/8	–	sil	2, m, gr	lo	3f
Ap2	16–25	cs	5Y 5/1	–	c, 2, f, 5Y 5/1	sicl	cdy	vfi	1f
Btg1	25–50	gs	5Y 5/1	m, 3, p, 10YR 6/6	c, 2, d, 5Y 7/1	sicl	3, c, sbk	fi	–
Btg2	50–70	gs	5Y 5/1	m, 3, p, 7.5YR 6/6	c, 2, d, 5Y 7/1	sicl	3, c, sbk	fi	–
Btg3	70–85	aw	5Y 5/1	m, 3, p, 7.5YR 6/6	c, 2, d, 5Y 7/1	sicl	3, c, sbk	fi	–
Ab	85–100	aw	10YR 3/1	–	–	sicl	2, m, sbk	fi	–
Bb	100–120	–	5Y 5/1	m, 3, d, 10YR 7/8	–	sicl	3, c, sbk	fi	–

Table S1 Description and classification of the studied soil profiles. [a] Soil horizon boundary distinctness: a, abrupt; c, clear; g, gradual. Soil horizon boundary: s, smooth; w, wavy; [b] Redoximorphic feature quantity: f, few; c, common; m, many; Size: 1, fine; 2, medium; 3, coarse. Contrast; p, prominent; d, distinct; f, faint; [c] Soil texture: sil, silt loam; sicl, silty clay loam; [d] Soil structure grade: 1, weak; 2, moderate; 3, strong. Size: f, fine; m, medium; c, coarse. Type: gr, granular; sbk, subangular blocky; cdy, cloddy; ma, massive; [e] Consistence: lo, loose; fr, friable; fi, firm; vfi, very firm; [f] Roots quantity: 1, few; 2, common; 3, many. Size: f, fine; [g] WRB = World referece base for soil resources[1]; ST = Soil Taxonomy[2]; CST = Chinese Soil Taxonomy[3].

Horizon	Depth (cm)	Boundary [a]	Munsell color (dry)	Redoximorphic features [b]		Texture [c]	Structure [d]	Consistence (moist) [e]	Roots [f]
				Soft masses	Depletions				
**P0-RC: Uncultivated soil (time zero); classification [7]: Plinthic Ferralsols (WRB), Aquults (ST), Udic Ferrosols (CST)**									
A	0–12	gs	10R 5/6	–	–	cl	3, f, gr	fr	3f
Br1	12–47	gs	10R 5/8	–	–	cl	3, f, gr	fr	1f
Br2	47–87	gs	10R 5/8	–	–	cl	3, f, gr	fr	1f
C	87–120	gs	7.5R 4/8	–	–	cl	3, f, gr	fr	–
**P60-RC: 60-yr paddy soil; classification: Hydragric Anthrosols (WRB), Hapludalfs (ST), Hapi-Stagnic Anthrosols (CST)**									
Ap1	0–10	cs	10YR 5/2	f, 1, p, 2.5YR 5/8	–	sicl	2, f, gr	lo	3f
Ap2	10–18	cs	10YR 6/4	–	–	sicl	cdy	fi	1f
Br1	18–30	cs	10YR 6/4	f, 1, p, 10YR 6/6	–	cl	2, f, sbk	fi	–
Br2	30–60	gs	5YR 6/5	f, 1, p, 10YR 6/6	–	cl	2, f, sbk	fr	–
Br3	60–85	gs	10R 5/6	f, 1, p, 10YR 6/6	–	cl	2, f, sbk	fr	–
BC	85–120	gs	10R 5/7	f, 1, p, 10YR 6/6	–	cl	2, f, sbk	fr	–
**P150-RC: 150-yr paddy soil; classification: Hydragric Anthrosols (WRB), Hapludalfs (ST), Hapi-Stagnic Anthrosol (CST)**									
Ap1	0–11	cs	10YR 5/3	f, 1, p, 2.5YR 5/8	–	sicl	2, f, gr	lo	3f
Ap2	11–20	cs	10YR 6/3	–	–	sicl	cdy	fi	1f
Br1	20–28	cs	10YR 6/4	m, 1, p, 10YR 6/6	–	sicl	2, f, sbk	fi	–
Br2	28–35	gs	10YR 6/3	m, 1, p, 10YR 6/6	–	cl	2, f, sbk	fi	–
Br3	35–48	cs	10YR 6/4	m, 1, p, 10YR 6/6	–	cl	2, f, sbk	fr	–
BC	48–64	gs	10YR 6/4	m, 1, p, 10YR 6/6	–	cl	2, f, sbk	fr	–
C	64–120	gs	10YR 7/4						
**P300-RC: 300-yr paddy soil; classification: Hydragric Anthrosols (WRB), Hapludalfs (ST), Fe-leachi-Stagnic Anthrosol (CST)**									
Ap1	0–10	cs	5YR 6/1	m, 1, p, 2.5YR 5/8	–	sicl	2, f, gr	lo	3f
Ap2	10–22	cs	7.5YR 6/1	–	m, 1, f, 5Y 5/1	sicl	cdy	fi	1f
Br1	22–45	gs	7.5YR 5/3	–	m, 1, f, 5Y 5/1	sicl	2, m, sbk	fi	–
Br2	45–65	gs	7.5YR 6/3	m, 3, p, 10YR 6/6	m, 1, f, 5Y 5/1	sicl	3, m, sbk	fi	–
BC	65–120	as	7.5YR 6/4	m, 3, p, 10YR 6/6	m, 1, f, 5Y 5/1	sicl	3, m, sbk	fi	–

Continued Table S1 Description and classification of the studied soil profiles. [a] Soil horizon boundary distinctness: a, abrupt; c, clear; g, gradual. Soil horizon boundary: s, smooth; w, wavy; [b] Redoximorphic feature quantity: f, few; c, common; m, many; Size: 1, fine; 2, medium; 3, coarse. Contrast; p, prominent; d, distinct; f, faint; [c] Soil texture: sil, silt loam; sicl, silty clay loam; [d] Soil structure grade: 1, weak; 2, moderate; 3, strong. Size: f, fine; m, medium; c, coarse. Type: gr, granular; sbk, subangular blocky; cdy, cloddy; ma, massive; [e] Consistence: lo, loose; fr, friable; fi, firm; vfi, very firm; [f] Roots quantity: 1, few; 2, common; 3, many. Size: f, fine; [g] WRB = World referece base for soil resources[1]; ST = Soil Taxonomy[2]; CST = Chinese Soil Taxonom[3].

Horizon	Depth	BD [a]	Clay	pH	SOC [b]	$CaCO_3$	N_{tot} [c]	P_{tot}	K_2O	Na_2O	CaO	MgO	Fe_2O_3	Al_2O_3	MnO
	(cm)	$g\ cm^{-3}$	w%	(H_2O)	$g\ kg^{-1}$										
**P0-MS: Uncultivated soil (time zero) developed on marine sediment**															
C1	0-30	1.30	22.37	7.86	3.97	63.00	1.18	1.48	22.41	19.10	39.84	25.00	51.94	123.48	1.00
C2	30-60	1.30	21.70	7.90	4.96	59.64	1.12	1.49	22.44	17.35	38.65	24.31	50.69	122.17	0.94
C3	60-90	1.33	20.80	8.28	4.86	55.46	1.15	1.49	21.05	18.31	39.13	22.87	46.49	114.26	0.85
C4	90-120	1.33	20.57	8.24	5.33	55.04	1.16	1.51	20.64	18.95	44.23	22.13	44.58	109.07	0.80
**P50-MS: 50-yr paddy soil developed on marine sediment**															
Ap1	0-16	1.22	28.01	7.59	20.84	3.95	2.22	2.66	22.11	14.56	12.02	18.67	51.04	127.96	0.69
Ap2	16-25	1.56	24.17	8.49	5.17	26.50	1.32	1.77	21.95	14.72	21.53	22.15	50.80	127.05	0.95
Bg1	25-50	1.47	24.80	8.45	4.66	21.91	1.45	1.80	21.31	14.42	20.06	21.31	49.00	121.23	0.80
Bg2	50-70	1.47	26.20	8.05	3.04	30.26	1.28	1.56	23.22	14.84	21.59	23.29	53.78	132.69	1.13
Bg3	70-100	1.45	28.36	7.86	3.79	17.33	1.24	1.40	24.85	12.96	13.59	23.53	58.98	142.29	0.92
BCg	100-120	1.41	26.47	8.33	2.58	39.44	1.26	1.47	22.36	13.87	25.79	22.80	55.69	128.86	1.14
**P300-MS: 300-yr paddy soil developed on marine sediment**															
Ap1	0-17	1.15	24.53	6.51	24.73	0.41	2.61	2.11	20.05	15.25	9.56	15.23	46.63	118.95	0.55
Ap2	17-26	1.45	25.68	8.09	6.92	3.95	1.80	1.75	20.70	15.80	11.32	16.53	48.11	121.39	0.83
Bg1	26-43	1.63	31.00	8.35	2.79	26.91	1.60	1.44	23.79	13.81	18.36	23.58	56.17	136.85	0.95
Bg2	43-70	1.60	29.97	8.66	2.86	0.00	1.45	1.31	27.09	13.53	21.82	26.00	57.22	148.43	0.98
Bg3	70-90	1.44	34.65	8.54	2.78	40.68	1.46	1.38	26.62	13.17	26.97	25.89	57.71	145.92	1.49
BCg	90-120	1.40	32.78	8.63	2.37	40.72	1.50	1.47	24.21	14.98	30.59	23.79	49.86	131.59	1.23
**P700-MS: 700-yr paddy soil developed on marine sediment**															
Ap1	0-15	1.10	24.29	6.26	18.73	0.00	2.08	1.46	21.76	15.97	8.64	13.47	41.54	122.39	0.53
Ap2	15-22	1.46	24.54	6.85	7.40	0.00	1.60	1.19	22.80	16.92	8.75	14.16	42.57	126.29	0.67
Eg	22-42	1.49	25.51	7.45	3.89	0.00	1.48	0.99	23.34	16.61	7.70	14.03	43.56	129.91	0.92
Btg1	42-60	1.48	45.58	7.40	3.15	0.00	1.54	1.02	27.18	12.13	5.87	18.18	76.74	160.24	0.55
Btg2	60-90	1.46	49.80	7.51	3.05	0.00	1.35	1.21	28.51	11.14	5.50	19.62	79.83	166.08	0.52
Ab	90-112	1.42	28.05	7.83	12.30	0.00	1.30	0.95	23.76	15.92	8.66	13.37	31.26	131.98	0.30
Bb	112-120	1.49	30.56	8.00	2.71	0.00	1.28	0.94	25.67	15.22	6.71	16.70	46.94	141.60	0.35
**P1000-MS: 1000-yr paddy soil developed on marine sediment**															
Ap1	0-16	1.15	24.35	6.76	14.23	0.00	1.28	1.56	21.71	16.19	8.02	12.31	40.26	123.45	0.58
Ap2	16-25	1.56	44.54	7.57	5.65	0.00	1.18	0.66	27.14	11.90	6.32	17.57	57.90	162.48	0.64
Btg1	25-50	1.43	42.62	7.54	3.32	0.00	1.16	0.72	26.99	12.64	6.14	18.70	65.52	159.81	0.53
Btg2	50-70	1.30	42.93	7.59	3.71	0.00	1.15	0.94	27.31	12.38	6.44	18.88	74.89	156.61	0.66
Btg3	70-85	1.28	49.48	7.69	4.27	0.00	1.14	1.33	28.68	11.08	6.02	19.16	90.20	161.98	1.12
Ab	85-100	1.41	40.36	7.68	9.93	0.00	1.20	0.76	24.12	13.06	7.79	16.37	45.35	152.41	0.93
Bb	100-120	1.41	33.51	7.57	2.87	0.00	1.15	1.37	23.99	13.79	7.19	17.14	57.83	144.33	1.02

Table S2 Basic soil physico-chemical properties of the studied profiles. [a] bulk density; [b] soil organic carbon; [c] total concentration.

	Depth	BD [a]	Clay	pH	SOC [b]	$CaCO_3$	N_{tot} [c]	P_{tot}	K_2O	Na_2O	CaO	MgO	Fe_2O_3	Al_2O_3	MnO
**Horizon**	**cm**	**g cm^{-3}**	**w%**	**(H_2O)**	**g kg^{-1}**										
**P0-RC: Uncultivated soil (time zero) developed on Quaternary red clay**															
A	0–12	1.19	54.72	5.35	8.70	0.00	0.80	0.74	13.43	0.87	0.43	6.48	65.34	166.07	0.49
Br1	12–47	1.23	50.40	5.28	3.20	0.00	0.44	0.72	12.80	0.97	0.41	5.62	61.88	154.57	0.41
Br2	47–87	1.31	55.44	5.33	2.55	0.00	0.41	0.82	13.17	1.28	0.35	6.42	64.84	164.14	0.36
C	87–120	1.43	56.52	4.55	2.26	0.00	0.41	0.75	14.43	0.95	0.41	5.56	70.96	180.96	0.39
**P60-RC: 60-yr paddy soil developed on Quaternary red clay**															
Ap1	0–10	0.91	28.08	4.86	19.28	0.00	1.90	1.31	11.10	1.45	1.08	3.91	26.29	78.84	0.19
Ap2	10–18	1.65	39.42	5.64	5.10	0.00	0.62	0.90	11.62	1.36	1.55	4.25	52.94	87.28	0.57
Br1	18–30	1.56	43.20	6.13	4.08	0.00	0.53	1.03	13.56	1.31	2.04	5.43	50.42	111.42	2.53
Br2	30–60	1.43	56.88	6.14	3.92	0.00	0.57	1.13	17.37	1.33	2.03	8.67	62.02	154.38	1.03
Br3	60–85	1.46	54.36	6.19	2.70	0.00	0.52	0.91	17.18	1.27	1.58	6.84	59.47	152.34	0.83
BC	85–120	1.44	49.86	6.13	2.04	0.00	0.48	0.83	16.34	1.20	0.59	6.52	62.47	155.23	1.38
**P150-RC: 150-yr paddy soil developed on Quaternary red clay**															
Ap1	0–11	0.92	37.62	5.40	15.61	0.00	2.12	2.23	7.51	0.85	1.43	3.25	46.64	77.03	0.16
Ap2	11–20	1.62	39.06	5.61	12.29	0.00	0.85	2.12	7.26	0.87	1.14	2.92	49.05	74.62	0.16
Br1	20–28	1.56	37.62	6.09	11.81	0.00	0.47	1.31	8.61	0.88	1.53	3.62	51.99	92.13	0.40
Br2	28–35	1.48	46.44	6.18	4.22	0.00	0.36	0.90	8.94	0.85	1.58	3.88	55.31	95.99	0.55
Br3	35–48	1.44	47.34	6.10	4.25	0.00	0.46	0.75	9.08	0.82	1.88	4.26	52.35	104.32	0.49
BC	48–64	1.46	44.28	5.98	4.29	0.00	0.38	0.78	9.55	0.88	1.94	4.13	53.13	96.54	0.52
C	64–120	1.43	41.40	6.10	3.97	0.00	0.43	0.77	15.64	0.92	2.19	6.36	50.32	146.69	0.51
**P300-RC: 300-yr paddy soil developed on Quaternary red clay**															
Ap1	0–10	1.09	33.66	5.93	23.94	0.00	1.04	1.33	7.03	0.81	1.30	3.10	35.53	78.31	0.17
Ap2	10–22	1.60	33.30	5.86	18.97	0.00	0.45	0.92	8.47	1.23	1.66	3.97	39.89	100.52	0.45
Br1	22–45	1.68	37.98	5.96	7.86	0.00	0.47	0.58	8.66	0.83	1.56	3.96	61.80	100.61	0.43
Br2	45–65	1.50	37.98	6.25	5.74	0.00	0.43	0.80	9.40	0.80	2.19	4.70	57.78	120.84	0.95
BC	65–120	1.48	39.78	6.40	5.46	0.00	0.31	0.85	10.59	0.66	2.19	5.07	51.38	155.31	0.33

Continued Table S2 Basic soil physico-chemical properties of the studied profiles. [a] bulk density; [b] soil organic carbon; [c] total concentration.

Parameter	Measurement	Unit	Implications
Total Fe	HF-$HClO_4$ extraction	g kg^{-1}	Sum of pedogenic Fe oxides and Fe bound to primary silicate minerals
Weakly bound Fe	$C_2H_2O_4$-$C_2H_8N_2O_4$ extraction	g kg^{-1}	Sum of weakly bound, poorly crystalline and organic bound Fe
Oxide-bound Fe	Citrate-bicarbonate-dithionite (CBD) extracted Fe minus $C_2H_2O_4$-$C_2H_8N_2O_4$ extracted Fe	g kg^{-1}	Pedogenic crystalline Fe oxides during soil evolution
Silicate-bound Fe	HF-$HClO_4$ extracted Fe minus CBD extracted Fe	g kg^{-1}	Fe bound to primary silicate minerals
Magnetic susceptibility (χ_m)	Measured at both low (0.47 kHz, χ_{lf}) and high frequencies (4.7 kHz, χ_{hf})	10^{-8} m^3 kg^{-1}	χ_m reflects the concentration of all magnetic minerals[4]
Saturation isothermal remanent magnetization (SIRM)	Isothermal remanent magnetization (IRM) measured at 1000 mT	10^{-4} A m^2 kg^{-1}	In contrast to χ_m, SIRM is not affected by paramagnetic (e.g., lepidocrocite and ferrihydrite) or diamagnetic (e.g., quartz) minerals. SIRM reflects the concentration of ferrimagnetic (magnetite and/or maghemite) and antiferromagnetic minerals (hematite and/or goethite)[4]
Soft isothermal remanent magnetization (IRM_s)	$0.5 \times (SIRM - IRM_{-20\ mT})$	10^{-4} A m^2 kg^{-1}	IRM_s reflects the low-coercivity ferrimagnetic minerals (magnetite and/or maghemite) if the *L*-ratio fluctuates significantly[4-6]
Hard isothermal remanent magnetization (IRM_h)	$0.5 \times (SIRM + IRM_{-300\ mT})$	10^{-4}A m^2 kg^{-1}	IRM_h reflects the weakly magnetic but high-coercivity antiferromagnetic minerals (hematite and or goethite) if the *L*-ratio is relatively constant[4-6]
Frequency magnetic susceptibility (χ_d)	$[(\chi_{lf} - \chi_{hf})/\chi_{lf}] \times 100\%$	%	χ_d reflects the concentration of a narrow particle size window of ultrafine superparamagnetic (SP, 0.012~0.022 μm) grains in soils that includes most pedogenically formed ferrimagnetics[4, 7]
Anhysteretic remnant magnetization (ARM)	Determined at 0.04 mT imposed on an AC field with decreasing amplitude from 100 mT to 0 mT	10^{-6} A m^2 kg^{-1}	ARM is mainly carried by single-domain (SD) and small pseudo-single domain (PSD) ferrimagnetic particles[4-6]
S-ratio	$0.5 \times [(SIRM - IRM_{-300\ mT}) / SIRM]$	%	*S*-ratio provides a measure of the relative amounts of low-coercivity to high-coercivity remanence[4]
L-ratio	$IRM_h / [0.5 \times (SIRM + IRM_{-100\ mT})]$	%	The ratio of two residual remanences after AF demagnetization of an IRM imparted in a 1 T field with a peak AF at 100 mT and 300 mT[6]

Table S3. Interpretations of the operationally defined Fe pools and measured magnetic parameters.

References

1. FAO. World Reference Base for Soil Resources 2006 (World Soil Resour. Rep. 103. FAO, Rome, 2006).
2. Soil Survey Staff. Keys to Soil Taxonomy. 11th edn (NRCS, Washington, DC, 2010).
3. Cooperative Research Group on Chinese Soil Taxonomy. Chinese Soil Taxonomy (Science Press, Beijing, 2001).
4. Evans, M. E. & Heler, F. Environmental Magnetism (Academic Press, San Diego, CA, 2003).
5. Lu, S. G. Soil Magnetism and Environment in China (Higher Education Press, Beijing, 2003).
6. Liu, Q. S., Roberts, A. P., Torrent, J., Horng, C. S. & Larrasoaña, J. C. What do the HIRM and S-ratio really measure in environmental magnetism? Geochem. Geophy. Geosy. 8, Q09011; 10.1029/2007GC001717 (2007).
7. Dearing, J. A., Dann R. J. L., Hay, K., Lees, J. A., Loveland, P. J., Maher, B. A. & O'Grady, K. Frequency-dependent susceptibility measurements of environmental materials. Geophys. J. Int.124, 228–240 (1996).
